# The role of E26 transformation-specific variant transcription factor 5 in colorectal cancer cell proliferation and cell cycle progression

**DOI:** 10.1038/s41419-021-03717-5

**Published:** 2021-04-30

**Authors:** Yi Peng, Haoran Feng, Changgang Wang, Zijia Song, Yaqi Zhang, Kun Liu, Xi Cheng, Ren Zhao

**Affiliations:** 1 Department of General Surgery, Ruijin Hospital, Shanghai Jiao Tong University School of Medicine, 200025 Shanghai, China; 2 Shanghai Institute of Digestive Surgery, Ruijin Hospital, Shanghai Jiao Tong University School of Medicine, 200025 Shanghai, China

**Keywords:** Cancer therapeutic resistance, Oncogenes

## Abstract

E26 transformation-specific variant transcription factor 5 (ETV5) contributes to tumor growth and progression and promotes colorectal cancer (CRC) angiogenesis. Previous studies indicate that ETV5 may regulate the cell cycle, but its detailed mechanism remain unclear. Gene Ontology (GO) analysis of RNA-seq data revealed that ETV5 possibly regulates the cell cycle in CRC. Here, in vitro and in vivo experiments were performed to verify that ETV5 promoted tumor progression and influenced cell cycle G1/S transition. Cell cycle PCR array and co-immunoprecipitation (Co-IP) helped identify the p21-CDKs pathway. Chromatin immunoprecipitation (ChIP) and luciferase reporter assays were performed to determine whether ETV5 binds to the p21 promoter. ETV5 and p21 were detected by immunohistochemistry, and the effects of their expression on CRC patients were evaluated. ETV5 upregulation enhanced tumor proliferative capacity and promoted G1 phase transfer to the S phase. ETV5 knockdown slowed the growth of CRC cells and repressed the G1/S transition. We also found p21 as a downstream target of ETV5. p21 knockdown resulted in faster CRC cell growth and in more cells being driven from the G0/1 phase into the S phase. Co-IP experiments showed that p21 banding to CDK2, CDK4, and CDK6 inhibited p130 phosphorylation. Using the ChIP and luciferase reporter assay, we confirmed that ETV5 bound to the p21 promoter and repressed p21 expression. CRC patients with high ETV5 expression and low p21 expression showed the worst prognosis. Finally, by targeting p21 to regulate CDK function, ETV5 also changed drug-sensitivity to palbociclib and dinaciclib. In conclusion, ETV5 promoted cell cycle G1/S transition through transcriptional inhibition of p21, thereby accelerating tumor growth. Moreover, ETV5 changed drug-sensitivity to palbociclib and dinaciclib. Therefore, therapeutic regimens targeting ETV5 may be promising in improving the efficacy of target-CDK treatment in CRC.

## Introduction

Colorectal cancer (CRC) represents 10.2% of all new cancer cases, and is the third most prevalent cancer worldwide. It is also the second most common cause of cancer mortality, and in 2018, led to >800,000 cancer deaths worldwide^1^. Increasing attention has been focused on cancer prevention. With advancing therapeutic strategies in surgery, radiation therapy, targeted therapy, and chemotherapy, the overall survival rate has improved^2–4^. Deregulation of the cell cycle underlies the aberrant cell proliferation that characterizes cancer, and as DNA replication is closely related to the cell cycle, DNA damage is a critical determinant of whether the cell cycle loses control and develops cancer, leading to most chemotherapy drugs targeting the DNA^5,6^. Because of the fundamental relationship between cell cycle and cancer, the detailed mechanism in cancer cell cycle regulation requires further elucidation.

The E26 transformation-specific (ETS) transcription factors consist of 28 family members in humans and can be divided into 12 subfamilies (e.g., ETS, ERG, ELF, and PEA3) according to sequence similarity and location of the ETS domain^7^. The ETS family regulates the expression of genes involved in normal cell development, proliferation, differentiation, angiogenesis, and apoptosis. Dysregulation of these transcription factors facilitates cell proliferation in cancers, and several members participate in invasion and metastasis by activating certain gene transcriptions^8^. ERG aberrant expression occurs in 50% of prostate tumors, and that of ETS family transcription factors ETV1 and ETV4 occurs in another 10% of cases. These three ETS factors are thought to promote tumorigenesis in the majority of prostate cancers^9^. ETS-1 transcription factor overexpression in breast cancers is associated with invasive features and promotes angiogenesis by creating a paracrine pro-invasive environment for endothelial cells, leading to a poor prognosis in patients^10^. Moreover, ERG and ETV6 promote thrombocytopenia and leukemia and are crucial for its maintenance^11,12^.

ETS variant transcription factor 5 (ETV5), also called ERM, belongs to the PEA3 subfamily (ETV1, ETV4, and ETV5), was first reported to have a potential role in the regulation of breast cancer growth and progression^13^. More research has found that ETV5 participates in tumor oncogenesis and progression^14,15^. In addition, ETV5 plays a crucial role in epithelial-to-mesenchymal transition, resulting in the acquisition of migratory and invasive capabilities in different cancer cell lines^16–18^. In our previous study, we concluded that ETV5 overexpression stimulated proliferation and angiogenesis both in vitro and in vivo, and a mechanism study found that ETV5 activates angiogenesis in CRC tumors through transcriptional upregulation of PDGF-BB^19^. Furthermore, the RNA-seq data from our last study indicated that ETV5 was significantly related to cell cycle after gene ontology (GO) analysis. Previous studies support that ETV5 promotes tumor proliferation, but cell cycle changes have not yet been described in detail. Therefore, we decided to confirm and explore the specific relationship between ETV5 and the cell cycle. The cell cycle is a process in which a cell duplicates itself and divides into two cells. As a complex sequence of events, the full cell cycle process involves many regulatory proteins for proper cellular reproduction, including cyclin proteins and cyclin-dependent kinases, oncogenes and tumor-suppressor genes, and mitotic checkpoint proteins^20,21^. In the process of copying itself, cellular DNA damage causes cancer. However, DNA damage is also the target of most cancer therapies such as radiation therapy and many chemotherapeutic agents^6^. Since the cell cycle process involves many regulatory proteins, the study of the tumor cell cycle can provide a clear rationale for targeting these proteins in cancer treatment.

In this study, GO analysis of RNA-seq data indicated that ETV5 was significantly related to the cell cycle. Through in vivo and in vivo experiments, we confirmed that ETV5 upregulation enhanced tumor proliferative capacity. Flow cytometry showed that ETV5 can promote G1 phase transfer to the S phase. Moreover, ETV5 knockdown notably slowed CRC cell growth and repressed the G1/S transition. We also found p21 as a downstream target of ETV5. Co-immunoprecipitation (Co-IP) experiments showed that p21 banding to CDK2, CDK4, and CDK6, inhibited the phosphorylation of p130, and suppressed the G1/S transition. Using the chromatin immunoprecipitation (ChIP) and luciferase reporter assays, we confirmed that ETV5 bound to the p21 promoter and repressed p21 expression. Furthermore, by targeting p21 to regulate CDK function, ETV5 also changed the drug-sensitivity of some small-molecule CDK inhibitors such as palbociclib and dinaciclib. Finally, clinical data revealed that CRC patients with high ETV5 expression and low p21 expression showed the worst prognosis. These results indicate that ETV5 is a new potential diagnostic and prognostic marker in CRC and provides a reference for CDK inhibitor therapy in cancer.

## Results

### ETV5 enhanced tumor proliferative capacity and promotes G1/S transition

**Fig. 1:**
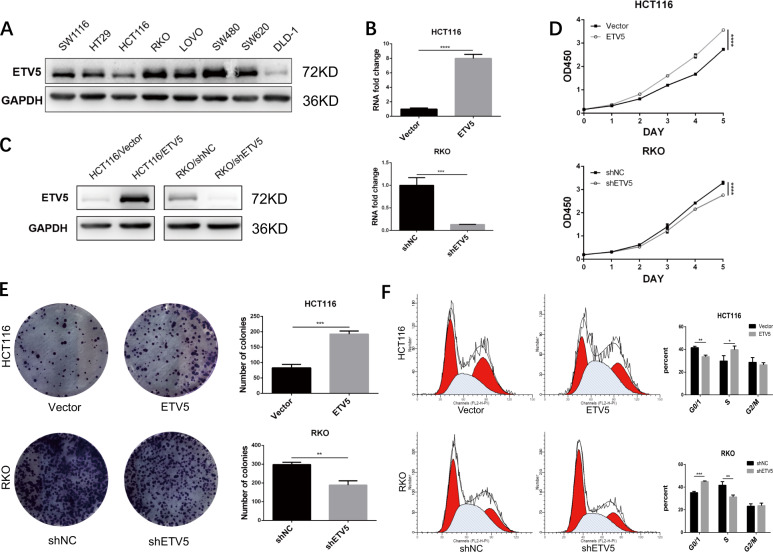
ETV5 enhanced tumor proliferative capacity and promoted G1/S transition. **A** ETV5 expression level in eight CRC cell lines, detected by Western blot. **B**, **C** The effect on ETV5 expression of shRNA knockdown in RKO and overexpression in HCT116. **D** CCK-8 assay of indicated sublines. **E** Colony formation assay. **F** Cell cycle analysis by flow cytometry to test the changes in HCT116 and RKO cell lines after ETV5 overexpression or knockdown. *p < 0.05, **p < 0.01, ***p < 0.001, ****p < 0.0001.

**Fig. 2:**
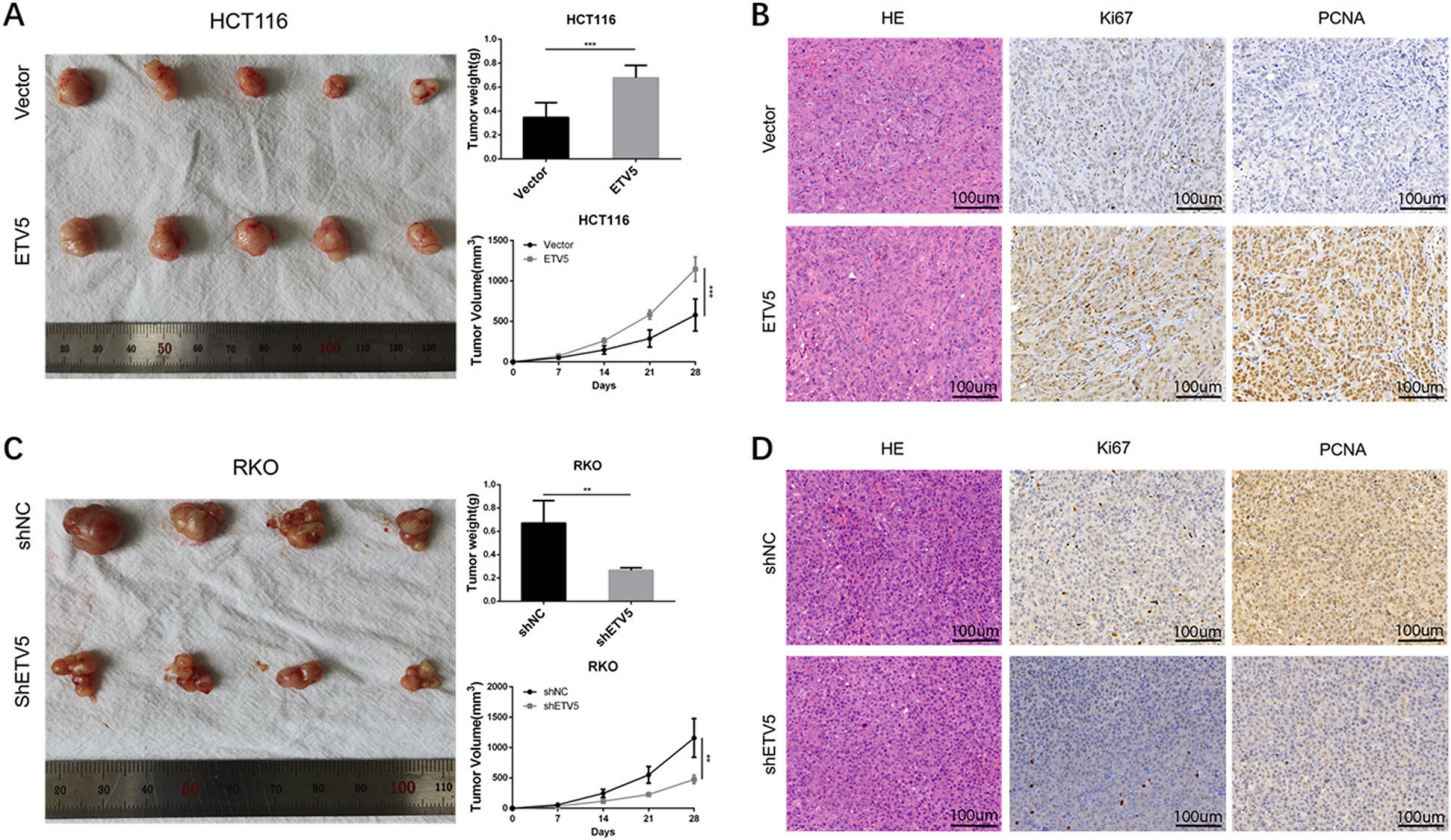
ETV5 promoted tumor proliferation in vivo. **A** and **C** Morphological observation and average volumes of xenografts of tumors formed after injecting nude mice with HCT116 cell lines overexpressing ETV5 and RKO cell lines with silenced ETV5(tumor volume at indicated time points, tumor weight at endpoint). **B** and **D** Representative images of H&E stain and IHC (Ki67 and PCNA) of xenografts of tumors. **B** Formed by HCT116 cell lines. **D** Formed by RKO cell lines. *p < 0.05, **p < 0.01, ***p < 0.001, ****p < 0.0001.

Based on the GO analysis of RNA-seq data from our last study^19^, we found that ETV5 was related to cell cycle regulation via many pathways (Supplement Fig. 1). To identify the functional role of ETV5 in cell cycle progression, we chose HCT116 and RKO from eight different CRC cell lines, as HCT116 cells have low ETV5 expression levels, whereas RKO cells have high ETV5 expression levels (Fig. 1A). We then knocked down ETV5 expression using lentivirus-mediated shRNA in RKO cells and ectopically overexpressed ETV5 in HCT116 cells, and verified the changes in ETV5 expression by quantitative reverse transcription PCR (RT-qPCR) and western blotting (Fig. 1B, C). The CCK-8 assay showed that ETV5 overexpression significantly promoted cell proliferation, whereas knocked down ETV5 clearly inhibited cell proliferation (Fig. 1D). The colony formation assay also confirmed that ETV5 promotes tumor proliferation (Fig. 1E). Cell cycle analysis showed that ETV5 overexpression significantly decreased the proportion of cells in the G1 phase and increased the proportion of cells in S phase, while knocked down ETV5 in RKO demonstrated consistent results (Fig. 1F). In vivo, cells were inoculated subcutaneously into the flank of nude mice and grown for 28 d. ETV5 overexpression resulted in larger tumors (Fig. 2A, *p* = 0.0010) and the tumors became smaller when we knocked down ETV5 (Fig. 2C, *p* = 0.0057). Proliferation was assessed using the IHC of Ki67 and PCNA, both of which showed positive regulation with ETV5 (Fig. 2B, D). Given the findings described above, we can conclude that ETV5 plays an oncogenic role and facilitates the G1/S transition in CRC cells.

### ETV5 regulate tumor growth and cell cycle via p21

**Fig. 3:**
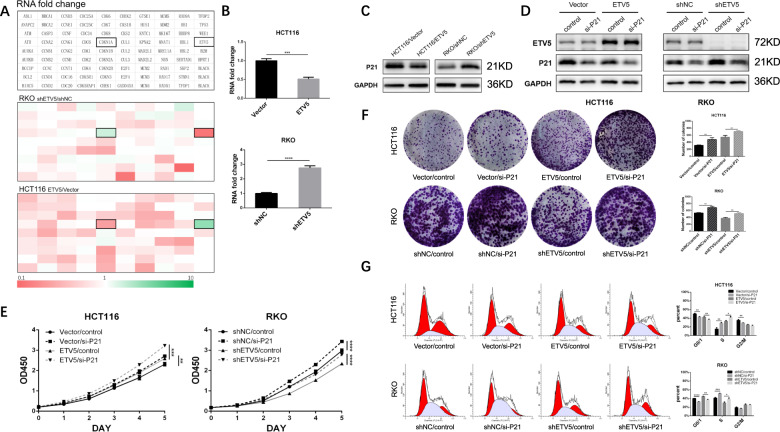
ETV5 regulated tumor growth and cell cycle via p21. **A** Cell cycle PCR array shows mRNA relative expression of 86 cell cycle related genes and ETV5. **B** and **C** RT-qPCR and western blot were performed to verify the impact of ETV5 on p21 expression. **D** Endogenous p21 was knocked down after transfection with siRNA. **E** and **F** CCK-8 assay and Colony formation assay after p21 silencing by siRNA. **G** Flow cytometry was performed again after silencing p21 in HCT116 and RKO cell lines. *p < 0.05, **p < 0.01, ***p < 0.001, ****p < 0.0001.

The MAPK pathway was checked yet we did not find a presumptive change that could explain the cell cycle G1/S phase transition. Then, cell cycle PCR array was performed to examine the expression of genes that participate in cell cycle regulation, and we found that CDKN1A, also called p21, had a negative relationship with ETV5 (Fig. 3A). RT-qPCR and western blotting verified that ETV5 had a negative impact on p21 expression (Fig. 3B, C). As a product of CDKN1A, a well-established cyclin-dependent kinase (CDK) inhibitor, p21, has been reported to play an important role in controlling cell cycle progression^22^. p21 is usually defined as an inhibitor of cell cycle progression owing to its ability to inhibit the activity of cyclin-dependent kinase (CDK)-cyclin complexes and PCNA^23^. We silenced p21 this way using small-interfering RNA (siRNA), which resulted in the cell proliferation ability being significantly increased (Fig. 3D, F). Flow cytometry was again performed, and we found that the cells showed an obvious transition from the G0/1 phase to the S phase (Fig. 3G). Therefore, the results above indicated that ETV5 could promote CRC cell proliferation and G1/S transition by inhibiting p21 expression.

### P21 binds to CDKs and change the phosphorylation of p130

**Fig. 4:**
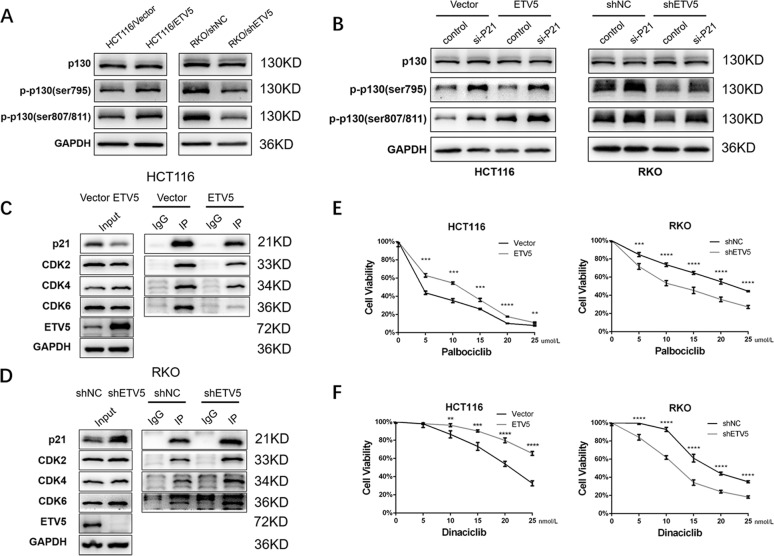
p21 was bound to CDKs and changed the phosphorylation of p130. **A** Effects of ETV5 overexpression and knockdown on p130 phosphorylation in HCT116 and RKO cells, detected by western blot. **B** Endogenous p21 was knocked down after transfection with siRNA in both HCT116 and RKO cell lines. **C** and **D** Co-IP was performed to examine whether p21 interacted with CDK2, CDK4, and CDK6 in both HCT116 and RKO cell lines. **E** and **F** CCK-8 assay was performed to test cell viability with the CDK inhibitors Palbociclib and Dinaciclib. *p < 0.05, **p < 0.01, ***p < 0.001, ****p < 0.0001.

Previous studies have reported that p21 inhibits cell cycle progression through the inhibition of CDK activity, such as that of CDK2 and CDK4, especially CDK2, which is required for the phosphorylation of the retinoblastoma (RB) protein with the consequent release and activation of E2F-dependent gene expression^24,25^. Some other experimental evidence, however, suggested that CDK2 is dispensable for cell cycle inhibition by p21 (ref. ^26,27^). Considering this, we tested our cells and found that ETV5 promoted the phosphorylation of p130, which belongs to the RB family (Fig. 4A, B). Co-IP was also conducted, and the results showed that p21 could combine CDK2, CDK4, and CDK6. p21 increased when ETV5 was knocked down and bound more CDKs, and reverse outcomes were observed in ETV5-overexpressing cells (Fig. 4C, D). Moreover, we used Palbociclib, a highly selective CDK4/6 inhibitor, to dispose of the cells. Results showed that cell viability increased when ETV5 was overexpressed in the HCT116 cell line and that cell viability was reduced after knockdown of ETV5 in the RKO cell line (Fig. 4E). Another inhibitor, Dinaciclib, exhibited results consistent with Palbociclib (Fig. 4F). Therefore, ETV5 can affect CDK function via p21, and can change the drug-sensitivity of Palbociclib and Dinaciclib in CRC cells.

### ETV5 suppress the expression of p21 by a p53-independent pathway

**Fig. 5:**
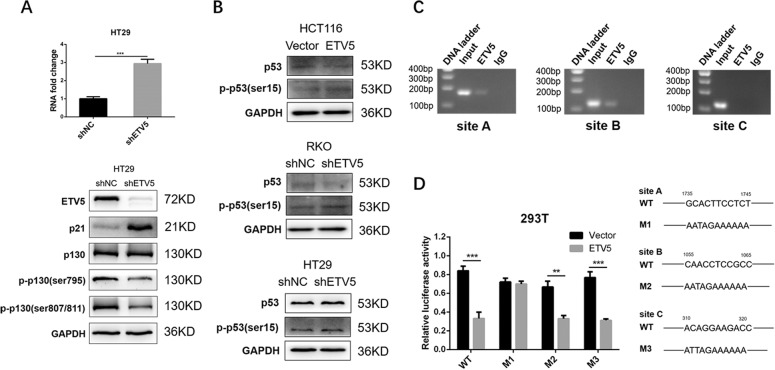
ETV5 suppressed the expression of p21 by a p53-independent pathway. **A** RT-qPCR and western blotting were performed to detect the changes of mRNA and protein in p21 and phosphorylation of p130 in HT29 cell lines expressing the mutant p53 gene. **B** Effects of ETV5 overexpression and knockdown on p53 expression and the phosphorylation of p53 in HCT116, RKO, and HT29 cell lines, detected by western blot. **C** Amplification of p21 promoter regions (a, b, and c) after ChIP using an ETV5 antibody on the 293 T cell line. **D** Luciferase reporter assay of three p21 promoter region mutations in the 293 T cell line. A specially designed plasmid was used to overexpress ETV5 in the 293T cell line. *p < 0.05, **p < 0.01, ***p < 0.001, ****p < 0.0001.

Previous research revealed that p21 mediates p53-dependent G1 growth arrest; however, updated studies have shown p53-independent pathways, leading to p21 induction^23,28,29^. The p21 gene was the first to be identified as being induced by the wild-type p53 protein; in contrast, no p21 induction was observed in the mutant p53-expressing cell line^30,31^. Moreover, RNA-seq data indicated that ETV5 may regulate cell cycle by the p53 pathway (Supplement Fig. 1). Considering that both RKO and HCT116 cell lines express the wild-type p53 protein, we selected a mutant p53 CRC cell line—HT29—which has high p53 expression levels, and knocked down ETV5. In HT29 cells, we found that p21 remarkably increased in both mRNA and protein, similar to the RKO cell line, and that phosphorylation of p130 also improved after ETV5 knockdown (Fig. 5A). Western blot results showed that p53 expression was low in RKO and HCT116 cells but was high in HT29 cells; moreover, the phosphorylation of p53 at the ser15 site did not change in any of the three cell lines (Fig. 5B). These results indicate that ETV5 regulates the expression of p21 via a p53-independent pathway. Therefore, we determined whether p21 was a direct transcriptional target of ETV5 using the ChIP assay. We surveyed sequences upstream of p21 ranging from −2000 bp to 0 bp with respect to the transcription start site and identified three putative ETV5 binding sites (details in Supporting Information Table S4). ChIP-PCR results showed that ETV5 could bind directly to sites A and B (Fig. 5C). Furthermore, a luciferase reporter assay using 293 T cells and plasmid with overexpressed ETV5 confirmed that the inhibition of p21 expression disappeared after the addition of the mutant site A sequence (Fig. 5D). Collectively, these findings validate that p21 is a direct transcriptional target of ETV5.

### ETV5 has a negative correlation with p21 in CRC tissues and predicts poor prognosis among CRC patients

**Fig. 6:**
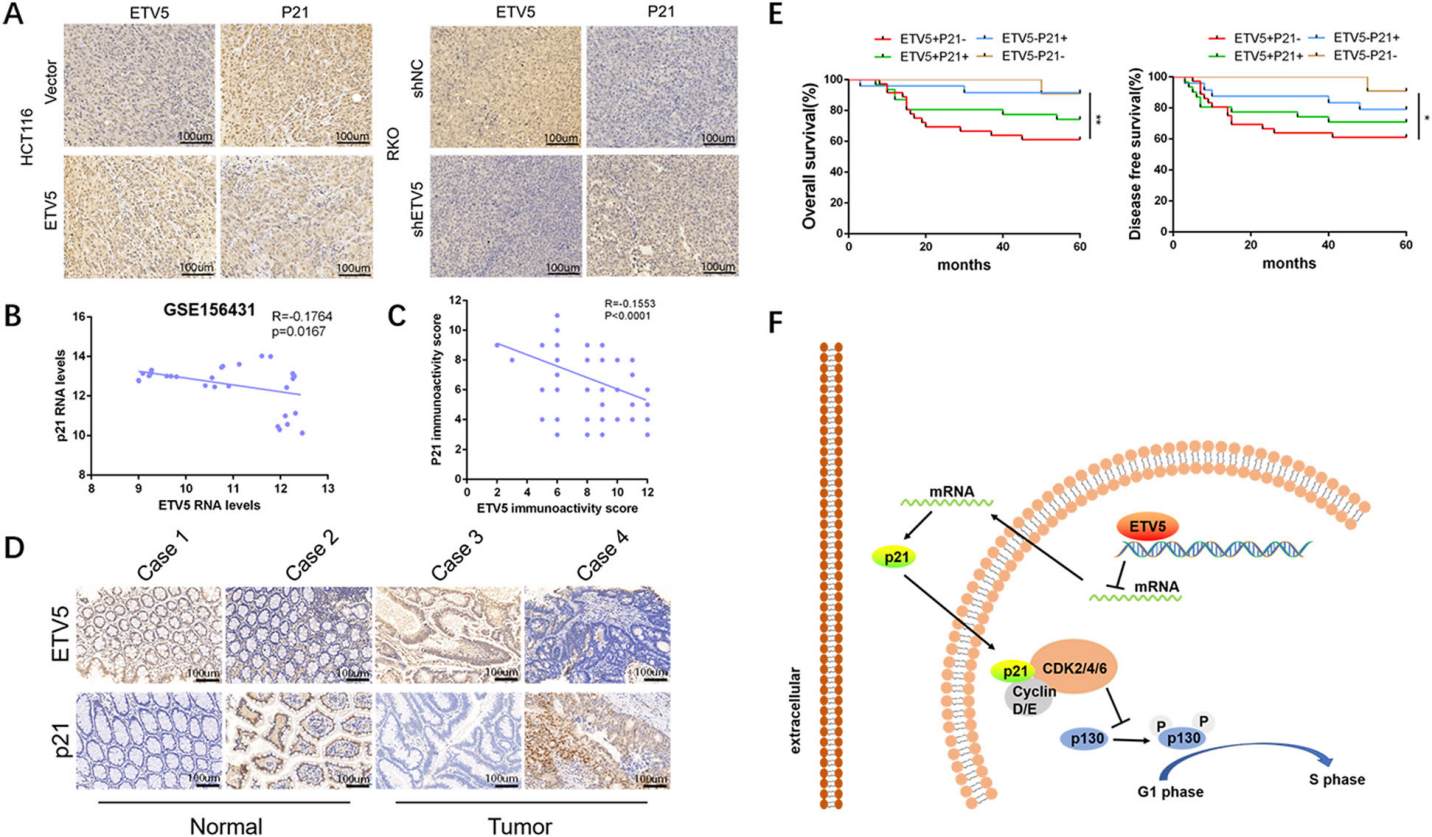
ETV5 had a negative correlation with p21 in CRC tissues and predicts poor prognosis among CRC patients. **A** The representative IHC staining images of ETV5 and p21 was performed on slices from a xenograft tumor model. **B** In GEO databases, ETV5 RNA level was significantly related to the p21 RNA level (GSE156431, *R* = −0.1764, *p* = 0.0167). **C** and **D** Negative correlation between ETV5 expression and p21 expression according to the statistical result of H-score (*p* < 0.0001). **E** Kaplan–Meier survival analysis revealed that patients with high ETV5 expression and low p21 expression exhibited the worst OS and DFS (*p* = 0.0048 and *p* = 0.0325, respectively). **F** Schematic of ETV5 function and mechanism in CRC cell cycle regulation, whereby ETV5 promoted G1/S transition by directly targeting p21. *p < 0.05, **p < 0.01, ***p < 0.001, ****p < 0.0001.

To reveal the relationships between ETV5 and p21 in vivo, p21 IHC was performed on slices from a xenograft tumor model. The results showed a remarkable negative correlation between ETV5 and p21 (Fig. 6A). In GEO databases, ETV5 RNA level was significantly related to p21 RNA level (*p* = 0.0167, Fig. 6B). Furthermore, the IHC of p21 and ETV5 was performed on 102 CRC tissues and we found a negative correlation between ETV5 expression and p21 expression according to the statistical results of H-score (*p* < 0.0001, Fig. 6C, D). These results prompted us to stratify patients based on the expression patterns of the two proteins and compare clinical outcomes. We found that patients with tumors and having high ETV5 expression and low p21 expression exhibited the worst OS and DFS (*p* = 0.0048 and *p* = 0.0325, respectively, Fig. 6E). Therefore, we concluded that ETV5 targeting inhibited the expression of p21 to suppress the G1/S transition (Fig. 6F).

## Discussion

Cell cycle deregulation is a common feature of human cancer. Cancer cells frequently display unscheduled proliferation and genomic instability^32^. The abnormal expression of many cell cycle proteins changes the biological characteristics of cancer cells, and kinases involved in cell cycle checkpoint function also constitute potential therapeutic targets. Their inhibition compromises checkpoint function, causes excessive DNA damage, and eventually leads to apoptosis^6,21^. The ETS family contains transcription factors that are important regulators of tumor progression. ETV5, a member of the subfamily of PEA3 (ETV1, ETV4, and ETV5), has a prominent role in regulating the progression of human breast cancer and thyroid cancer^13–15^. Our last study also proved that ETV5 promotes proliferation and enhances angiogenesis of CRC cells both in vitro and in vivo, and plays a role in cell cycle regulation^19^. ETS family members have been implicated in controlling the cell cycle not only in normal mammary gland development but also in breast tumorigenesis^33^. Moreover, ETV5 has been identified as the most important upstream factor that regulates SSC self-renewal and spermatocyte meiosis^34^. Previous studies have also confirmed that ETV5 promotes tumor proliferation. However, whether ETV5 regulates the tumor cell cycle remains unclear.

In this study, we demonstrated that ETV5 promotes CRC progression and alleviates G1 arrest by inhibiting p21 expression. As a universal cell cycle inhibitor that promotes cell cycle arrest in response to a variety of stimulations, p21 is a direct p53-regulated target gene, and has been thought to mediate p53 tumor suppression^35,36^. However, a study by Datto et al.^37^ demonstrated that TGF-β also causes rapid transcriptional induction of p21, and this p21 induction by TGF-β is not dependent on wild-type p53 (ref. ^37^). Another study showed that mimosine can increase both p21 mRNA and protein levels and induce a p53-independent p21 pathway in cancer cells^38^. Basically, pathways found in previous discoveries mainly promote p21 expression. However, our research found a p53-independent pathway that directly inhibited the expression of p21.

In the present study, p21 regulates cell cycle arrest primarily by binding CDKs and suppressing the phosphorylation of RB proteins, which is regarded as a switch in the transformation from G1 phase to S phase^23,30^. PCNA, which plays critical roles in many aspects of DNA replication and replication-associated processes, is another factor that is inhibited by interacting with the carboxy-terminal of p21 (ref. ^39^). p21 also modulates apoptosis, induces senescence, maintains cellular quiescence in response to various stimuli^40^, and contributes to cancer suppression and therapy^41^. The RB protein is a tumor suppressor responsible for a major G1 checkpoint, blocking S phase entry. The RB family includes three members, Rb, p107, and p130, and all three regulate E2F and the cell cycle. Furthermore, Rb and p130 are mainly responsible for the repression of G1/S. CDK4 or CDK6 initially phosphorylates RB in the G1 phase of the cell cycle, and CDK2 phosphorylates RB in late G1, which releases the respective E2F family member from RB, leading to the activation of transcriptional targets to advance the cell cycle^42–45^.

In our study, CRC cell lines, including HCT116, RKO, and HT29, all express p130. In addition, Rb and p107 were not seen in the Western blot results. Our research showed that ETV5 can regulate the cell cycle by directly suppressing the transcription of p21 in the context of CRC, and then intensify the phosphorylation of p130. Considering that p21 could regulate the function of CDKs, we used palbociclib and dinaciclib to dispose the cells. Results showed that cell viability improved when ETV5 is overexpressed, which indicates that ETV5 can reduce the sensitivity of CDK inhibitors. Targeting CDK4/6 activity has long been considered a promising approach for cancer treatment. As a cyclin-dependent kinase 4/6 (CDK4/6) inhibitor, albociclib is an established treatment for estrogen receptor-positive breast cancer^46,47^. There are at least 180 completed or ongoing clinical trials testing CDK4/6 inhibitors across a broad portfolio of cancers^48^. Furthermore, albociclib and dinaciclib demonstrated strong anti-tumor activity against CRC^49,50^. Thus, ETV5 is a potential biomarker for predicting the sensitivity of CDK inhibitor treatment in CRC.

In conclusion, our data reveal a novel mechanism whereby ETV5 regulates the CRC cell cycle. These findings indicate that ETV5 is a new potential diagnostic and prognostic marker in CRC and that targeting signaling pathways controlled by ETV5 may be a promising strategy for CRC therapy.

## Materials and methods

### Patients

A total of 204 consecutive unselected CRC tissues were collected following a protocol from CRC patients who underwent radical surgery at the Ruijin Hospital from 2011 to 2013. Before surgery, none of the patients received any anti-tumor treatment. Clinical parameters, pathological data, overall survival (OS), and disease-free survival (DFS) were recorded. All tumors were histologically diagnosed by at least three pathologists. Tumor stage was determined according to the UICC TNM classification system. Patient information is illustrated in Table S1. All patients signed an informed consent form.

### Cell lines, cell culture, palbociclib, and dinaciclib

Human CRC cell lines, RKO, HT29, SW620, SW1116, SW480, and HCT116 were purchased from ATCC (Rockville, MD, USA). Human CRC cell lines LOVO and DLD-1 were obtained from the Shanghai Institute of Biochemistry and Cell Biology, Chinese Academy of Sciences. CRC cell lines RKO, HT29, SW620, SW1116, SW480, HCT116, and DLD-1 were cultured in high-glucose RPMI-1640 supplemented with 10% fetal bovine serum (FBS) and 1% penicillin-streptomycin at 37 °C with 5% CO_2_. LOVO was cultured in high-glucose DMEM supplemented as described above. Palbociclib, a highly selective CDK4/6 inhibitor, was purchased from SELLECK (Cat. No. S1579, USA). Dinaciclib, an effective selective CDK inhibitor that inhibits CDK2, CDK5, CDK1, and CDK9, was purchased from MedChemExpress (Cat. No. HY-10492, USA).

### Generation of gene overexpression and knockdown cells

Lentivirus for ETV5 overexpression and three shRNAs were purchased from Shanghai Bioegene Co., Ltd. Lentiviral particles were transduced into CRC cells according to the manufacturer’s instructions. Then, puromycin (4 µg/mL) was added to establish stably expressing CRC cell lines. The three targeted ETV5 sequences are illustrated in Table S2. Short-hairpin RNA (shRNA)1 had the strongest effect and was used for subsequent experiments.

### siRNA transfection

P21 siRNA and control siRNA (Bioegene, Shanghai, China), specifically targeting the genes, were transfected using Lipofectamine 3000 reagent (Cat. No. L3000075, Invitrogen, USA). CRC cells were seeded into 6-well plates at 80%–90% concentrations, and 1 mL of the medium was added to each well. Subsequently, 50 pmol siRNA was mixed with 5.0 μL of Lipofectamine 3000 in 0.2 mL of medium, and the mixture was added to the wells. The siRNA sequences are illustrated in Table S3. siRNA1 had the strongest effect and was used for subsequent experiments.

### Western blot analysis

The total protein for Western blotting was extracted from cancer cells using RIPA buffer supplemented with a Protease and Phosphatase Inhibitor Cocktail (Cat. No. P002. Ncm Biotech, China). The protein concentration was evaluated using a BCA Protein Assay Kit (Cat. No. 23225. Thermofisher, USA). In brief, 20 μg of protein was separated by 10% or 12.5% sodium dodecyl sulphate–polyacrylamide gel electrophoresis (SDS-PAGE) gels and was transferred to polyvinylidene fluoride membranes (Tanon, China). After blocking with 5% bovine serum albumin for 2 h, the membranes were incubated with primary antibodies: anti-GAPDH (Cat. No. 60004-1-Ig, Proteintech, China), anti-ETV5(Cat. No. ab102010; Abcam, Cambridge, UK), anti-p21(Cat. No. 2947S, CST, USA), anti-Rb(Cat. No. 9309T, CST, USA), anti-Phospho-Rb (Ser795) (Cat. No. 9301T, CST, USA), anti-Phospho-Rb (Ser807/811) (Cat. No. 8516T, CST, USA), anti-P53 (Cat. No. 2524S, CST, USA), and anti-Phospho-p53 (Ser15) (Cat. No. 9286T, CST, USA). After washing thrice with TBST, the membranes were incubated with secondary antibodies: HRP-conjugated goat anti-rabbit IgG (Cat. No. SA00001-2, Proteintech, China) and HRP-conjugated goat anti-mouse IgG (Cat. No. SA00001-1, Proteintech, China) for 2h at room temperature. Finally, the membranes were visualized using a chemiluminescence system (Bio-Rad) according to the manufacturer’s protocol. Three independent experiments were conducted under the same conditions.

### Co-immunoprecipitation

The Co-IP kit was purchased from Absin (Cat. No. abs955, China). Experiments were performed following the protocol. The mixture was then detected by Western blotting. The primary antibodies used were: anti-p21 (Cat. No. 2947 S, CST, USA), anti-CDK2 (Cat. No. 2546 T, CST, USA), anti-CDK4 (Cat. No. 12790 T, CST, USA), and anti-CDK6 (Cat. No. 3136 T, CST, USA).

### Cell viability and colony formation assays

After digestion with trypsin, 2000 cells were plated in 96-well plates per well (four biological replicates) and cultured in a 37 °C incubator with 5% CO_2_. Cell viability was determined at 0, 24, 48, 72, 96, and120 h. To measure cell viability, we added CCK-8 (Dojindo Molecular Technologies Inc.) to each well for 2 h and the absorbance was measured at 450 nm using a microplate reader. In the inhibitor test, we plated 10 000 cells per well and cell viability was determined at 48 h. For the colony formation assay, 1500 cells were plated per well in 6-well plates and cultured at 37 °C for 14 days. Colony formation was determined by staining with 0.1% crystal violet in methanol for 30 min. In the drug-sensitivity experiment, we plated 5000 cells per well and cell viability was determined at 48 h after adding inhibitors. Data were obtained from three independent experiments.

### Chromatin immunoprecipitation

ChIP assays were performed using the ChIP kit (Cat. No. 17-371, Merck, Germany) according to the manufacturer’s protocol. Protein and DNA were crosslinked in 1% formaldehyde, extracted by SDS lysis buffer, and sheared by sonication. An ETV5 antibody (Santa Cruz Biotechnology, Inc., SC-22807) was used for immunoprecipitation. After purification of the precipitated DNA, PCR was conducted. Predicted binding sequences and primers amplifying the p21 promoter are listed in Supporting Information Table S4.

### Flow cytometry assay

The cell cycle of cancer cells was assessed using propidium iodide (PI)/RNase staining buffer (Cat. No. 550825, BD Pharmingen, USA) using flow cytometry. In brief, 150 000 cells were plated in 6-well plates per well and treated with non-FBS medium after 12 h. Cells were cultured in non-FBS medium for 24 h to decrease the effect of the growth factor in the serum and then the cells were reactivated using a serum-containing medium for 24 h. After digestion with trypsin, the attached and floating cells were harvested and washed twice with ice-cold PBS. Then, the cells underwent overnight fixation with 75% ethyl alcohol at −20 °C. Afterwards, the alcohol was disposed and the cells were washed twice with ice-cold PBS. PBS was removed, 300 µL propidium iodide (PI) was added, and this was mixed well. The tubes were placed in the dark at 37 °C for 30 min. Finally, the cell cycle of the cancer cells was evaluated by flow cytometry using the FACSC alibur system (BD Biosciences, USA).

### Luciferase reporter assay

p21 promoter fragments were cloned into the pGL3 Basic vector. Luciferase activity was examined using a Firefly & Renilla Luciferase Reporter Assay Kit (Cat. No. MA0518, Meilunbio, China), following the manufacturer’s instructions. Mutations in the binding sequences are listed in Supporting Information Table S4.

### In vivo xenograft tumor model

All animal experiments in this study were approved by the Guide for the Care and Use Laboratory Animals of the Ruijin Hospital, Shanghai Jiaotong University School of Medicine. Twenty 4-week-old male nude mice (Institute of Zoology, Chinese Academy of Sciences) were enrolled in this study. First, 1 × 10^6^ CRC cells were subcutaneously injected and tumor size was measured every 7 days, calculated using the formula: V = *π*/6 × (*W*^2^ × *L*). Finally, all mice were euthanized 28 days later. Samples were removed for immunohistochemical analysis.

### RNA extraction and quantitative RT-PCR

Total RNA was isolated from CRC cells using RNA isolator Total RNA Extraction Reagent (Cat. No. R401-01, Vazyme, China) according to the manufacturer’s instructions. cDNA was synthesized using HiScript II Q RT SuperMix for qPCR (+gDNA wiper) (Cat. No. R223-01, Vazyme, China). Quantitative PCR was performed using ChamQ Universal SYBR qPCR Master Mix (Cat. No. Q711-02, Vazyme, China).

### Immunohistochemistry analysis

Immunohistochemistry (IHC) was performed as previously described^19^. Briefly, the paraffin sections were baked at 65 °C for 2 h. Then, paraffin was removed by xylene, and the sections were dehydrated in gradient alcohol. Endogenous peroxidase was also inactivated using 3% hydrogen peroxide for 15 min. Antigen retrieval was performed using 0.01 mol/L sodium citrate buffer (pH 6.0) by heating (100 °C) for 15 min. After blocking with goat serum solution for 45 min, the sections were incubated with primary antibodies at 4 °C overnight. Antibodies used for IHC included antibodies against ETV5 (No. ab102010; Abcam, Cambridge, UK), p21 (Cat. No. 8242T, CST, USA), Ki67 (Cat. No. 9027T, CST, USA), and proliferating cell nuclear antigen (PCNA) (Cat. No. ab15497, Abcam). After washing thrice with PBS, sections were incubated with biotin-labeled secondary immunoglobulin (1:100, DAKO, Glostrup, Denmark) for 1 h at room temperature. Finally, the sections were stained with diaminobenzidine (DAB, DAKO, Glostrup, Denmark) and were re-stained with hematoxylin at room temperature. To quantify the expression of these molecules, IHC scores were separately evaluated by two pathologists. Each sample was scored on a sliding scale according to the percentage of positive cells as previously described (0 = 0%, 1 = 1–20%, 2 = 21–50%, 3 = 51–80%, 4 = 81–100%) and the staining intensity^51^ (0 = negative, 1 = weak, 2 = moderate, 3 = strong). The two scores were multiplied to generate an immunoreactive score (IRS) ranging from 0 to 12. The IRS scores from two pathologists were averaged and rounded to the nearest whole number. For statistical analysis, cases were classified as either negative (IRS 0–6) or positive (IRS 7–12).

### Statistics

Statistical analyses were performed using GraphPad Prism 6.0 (Inc., La Jolla, CA, USA). A two-tailed unpaired Student’ s *t*-test was performed to evaluate the difference between two groups. Data are shown as the mean ± SD. Survival analysis was conducted using the Kaplan–Meier method, and differences in survival rate were calculated by log-rank analysis. The correlation analysis between two variables was analyzed by Pearson correlation analysis. *p*-values < 0.05 were considered statistically significant.

## Supplementary information

supplementary figure 1 legends

Supplementary Figure 1

Table S1

Table S2

Table S3

Table S4

## Data Availability

All the data generated or analyzed during this study are included in this published article.
