# Supplementary material for: The role of E26 transformation-specific variant transcription factor 5 in colorectal cancer cell proliferation and cell cycle progression
Source: Cell Death Dis. 2021 Apr 30;12(5):427. doi: 10.1038/s41419-021-03717-5 (PMC8087822; doi:10.1038/s41419-021-03717-5)
Supplement: Supplementary file 1 — supplementary figure 1 legends [file 41419_2021_3717_MOESM1_ESM.docx]

**Supplement Figure 1.** GO analysis using our previous RNA-seq data showed that ETV5 was related to cell cycle regulation via many pathways.
