# Supplementary figures and images for: The role of E26 transformation-specific variant transcription factor 5 in colorectal cancer cell proliferation and cell cycle progression
Source: Cell Death Dis. 2021 Apr 30;12(5):427. doi: 10.1038/s41419-021-03717-5 (PMC8087822; doi:10.1038/s41419-021-03717-5)

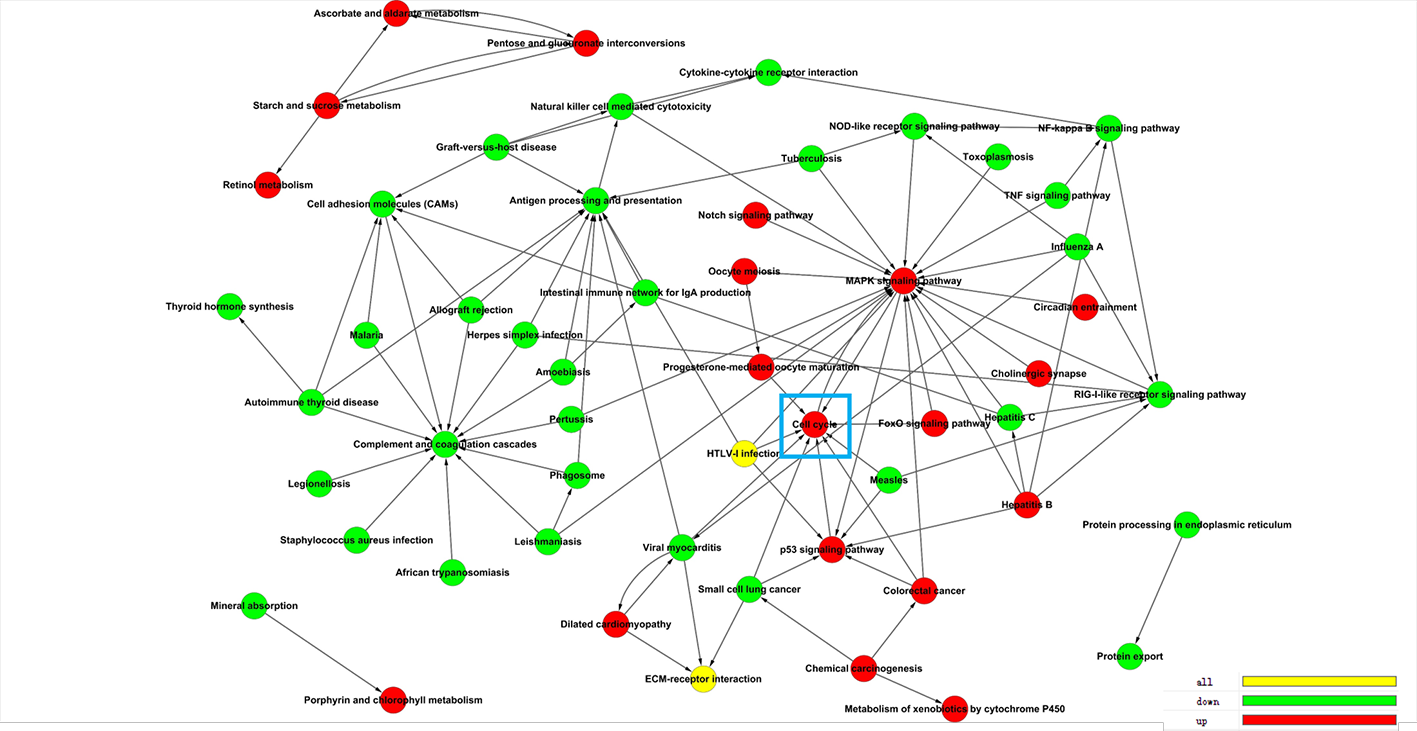

Supplement: Supplementary file 2 — Supplementary Figure 1 [file 41419_2021_3717_MOESM2_ESM.tif]
